# Supplementary material for: Highly Tunable Moiré Superlattice Potentials in Twisted Hexagonal Boron Nitrides
Source: Adv Sci (Weinh). 2024 Nov 8;12(4):2408034. doi: 10.1002/advs.202408034 (PMC11789588; doi:10.1002/advs.202408034)
Supplement: Supplementary file 1 — Supporting Information [file ADVS-12-2408034-s001.docx]

**Supporting Information**

**Highly tunable moiré superlattice potentials in**

**twisted hexagonal boron nitrides**

Kwanghee Han^1^, Minhyun Cho^1,2^, Taehyung Kim^1^, Seung Tae Kim^1^, Suk Hyun Kim^1,3^, Sang Hwa Park^4^, Sang Mo Yang^4^, Kenji Watanabe^5^, Takashi Taniguchi^6^, Vinod Menon^2^, Young Duck Kim^1,3*^

^1^Department of Physics, Kyung Hee University, Seoul, 02447, Republic of Korea.

^2^Department of Physics, City College and Graduate Center, City University of New York, New York, 305-0044, NY, USA.

^3^Department of Information Display, Kyung Hee University, Seoul, 02447, Republic of Korea.

^4^Department of Physics, Sogang University, Seoul, 04107, Republic of Korea.

^5^Research Center for Electronic and Optical Materials, National Institute for Materials Science, 1-1 Namiki, Tsukuba, 305-0044, Japan.

^6^Research Center for Materials Nanoarchitectonics, National Institute for Materials Science, 1-1 Namiki, Tsukuba, 305-0044, Japan.

*Corresponding author E-mail: ydk@khu.ac.kr

**Supporting Note 1. Details of twisted hBN devices**

**Table S1. List of Devices**

| Device Name | Structure | $d_{B}$^*^ (nm) | $d_{T}$^*^ (nm) | $d_{Ad}$^*^ (nm) |
| --- | --- | --- | --- | --- |
| Dev 1 (Fig. 1) | hBN/hBN | 3.5 | 1.7 | N.A. |
| Dev 2 (Fig. 2b-c) | hBN/hBN/hBN | 8.3 | 4.1 | 4.1 |
| Dev 3 (Fig. 2f-g) | hBN/hBN/hBN | 6.4 | 1.5 | 1.5 |
| Dev 4 (Fig. 3) | hBN/hBN/hBN | 6.8 | 16.5 | 17.3 |
| Dev 5 (Fig. 4 & S2) | hBN/hBN | 11.7 | 11.6 | N.A. |
| Dev 6 (Fig. S3a-b) | hBN/hBN | 14.8 | 13.9 | N.A. |
| Dev 7 (Fig. S3d-e) | hBN/hBN | 4.5 | 4.5 | N.A. |

(^*^$d_{B}$ : Bottom hBN thickness, $d_{T}$ : Top hBN thickness, $d_{Ad}$ : Additional top hBN thickness)

All the twisted hBN devices without bottom graphite (Dev 1, 5, 6, 7) are transferred on the SiO_2_/Si substrate. In this case, the Si backgate was used as an electrical ground for KPFM measurement. The other devices (Dev 2, 3, 4) used the bottom graphite as a ground to enhance the signal-to-noise ratio of KPFM signal. According to the equation (1) in main text, the electrical potential is different by the vertical distance to the interface^1^. Therefore, the moiré potential amplitudes of the samples are all different because each sample has a different hBN thickness and the height of the tip above the surface.

**Supporting Note 2. Electrical polarization by local stacking registry**

Figure S1a shows the top view of twisted hBN layers of BA stacking and its slightly displaced stacking to the in-plane direction. If we set the origin as AA stacking order ($r=0$), then the AB stacking and BA stacking have in-plane displacement of $r=1.45 Å$ and $r=2.9 Å$ respectively.

Figure S1b is the graph of normalized polarization by in-plane displacement $\boldsymbol{r}$. Recent theoretical study shows the electrical polarization as a function of in-plane displacement $\boldsymbol{r}$ between two boron atoms in adjacent layers.^1^ They calculated the differential charge $\Delta\rho$ with DFT method and it was fitted by

$P\left( \boldsymbol{r} \right)=\frac{P_{0}}{9}\left( f_{+}\left( \boldsymbol{r} \right)-f_{-}\left( \boldsymbol{r} \right) \right), f_{\pm}(\boldsymbol{r})\equiv\left| e^{i\boldsymbol{K}\cdot\boldsymbol{r}}+e^{i(\hat{C}_{3}\boldsymbol{K}\cdot\boldsymbol{r}\pm\frac{2\pi}{3})}+e^{i(\hat{C}_{3}^{2}\boldsymbol{K}\cdot\boldsymbol{r}\pm\frac{4\pi}{3})} \right|^{2}$, (1)

where K is the wavevector at the Brillouin zone corner, $\hat{C}_{3}$ is the rotation symmetry matrix. In AA stacking order, hBN layers of the twisted interface are well aligned. Therefore, no polarization is induced due to its out-of-plane mirror symmetry^2,3^. By contrast, AB stacking and BA stacking have maximum polarization values to opposite directions^1,2^.

Since the moiré potential of twisted hBN in Figure 4 of the main text was decreased, we assume that the transverse optical (TO) phonon by femtosecond laser can induce the in-plane displacements of AB/BA stacking. If the BA stacking is displaced $\pm0.60 \sim0.65 Å$, the moiré potential would be decreased to ~ 34.1 % according to Figure S1b. Therefore, we predict that the AB/BA stacking of twisted hBN is displaced about $\pm0.6 \sim0.65 Å$ after exposed to the femtosecond laser.

**Supporting Note 3. No topological defect or doping effect after femtosecond laser irradiation.**

Figure S2a shows an AFM topography of the twisted hBN in Fig. 4b of the main text. After being exposed to the deep UV femtosecond laser, we obtained the topography in the same area, which shows no hole or crack on the surface, as shown in Fig. S2b. We can see the same results from the cropped images in Fig. S2c and S2d. It is because the sample was exposed to the pulse laser with an intensity of 2.66 TW/cm^2^, which is much less than the laser-induced damage threshold intensity of 50 TW/cm^2^ ^4–6^. So, we confirm that there are no topological defects such as holes and cracks by comparing two topography images before and after laser irradiation.

Figure S2e is a KPFM result of the sample showing moiré superlattice. After femtosecond laser irradiation, we get a KPFM image of the same area in Fig. S2f. The KPFM technique offers visualization of local electric charges by detecting the surface potential difference which is related to the sample’s work function^7,8^. So it can detect a local doping effect of the sample^9–11^. By comparing two KPFM data, we verify no local doping effect in the twisted hBN sample after laser irradiation. Even though the moiré potential was diminished, there is no certain shape of surface potential change by irradiation. So, we confirmed that the origin of depolarization is not because of the topological damage or doping effect. Therefore, the deep UV femtosecond laser would be a useful tool for engineering moiré potential without any unwanted damage to the twisted vdW materials.

**Supporting Note 4. Additional statistical analysis of moiré potential engineering with femtosecond laser.**

In Figure S3a, there is a KPFM result of the sample in Fig. 4f of the main text. After deep UV femtosecond laser irradiation, the moiré pattern is almost invisible as shown in the Fig. S3b. To verify the tendency of decreasing the moiré potential amplitude same as the main figure, we calculated the normalized counts of the polarization strength. The average amplitude decreased from ~85.5 mV to ~28.0 mV which can be denoted as *ΔV* ~ 57.5 mV. The amplitude difference is well-matched with the line profile of Fig. 4h in the main text. Figures S3d and S3e show the KPFM images of another sample before and after laser irradiation. By analyzing the data of moiré potential distribution, there is a considerable depolarization with the amplitude change from ~ 87.2 mV to ~ 40.8 mV, which corresponds to *ΔV* ~ 46.4 mV. Since the two samples were exposed to the femtosecond pulse laser at the same intensity of 15.61 mJ/cm^2^, the magnitude of depolarization is similar. By investigating additional twisted hBN samples, we strongly support the reproducibility of depolarization after femtosecond pulse laser irradiation.

**Supporting Note 5. Local twist angle and shear strain difference analysis.**

The twist angle and strain differ slightly by location, even in the high-quality samples with regular moiré patterns. In the main text, we used the representative moiré patterns to compare two different twisted interfaces. The twist angle and moiré length are related by the equation as follows

$$\lambda_{m}\left( \theta\right)=a/2sin(\frac{\theta}{2})$$

where *a* is a lattice constant of hBN. For example, we extracted ~ 57 nm moiré length from Fig. S4b. According to the equation, the twist angle is $\sim0.25^{\circ}$. The same method can be used to analyze twisted angles from another moiré interface. To distinguish between two different interfaces, we analyzed the topography image. Since the sample in Fig. 2c. is partially covered by additional top hBN, we figure out that big moiré patterns are from the additional interface.

If the potential amplitude from each interface is significantly different, it might be useful to distinguish. However, the thickness of top hBN for most of the sample is not thick. So, the moiré length is more dominant variance to change the potential depth. Therefore, we only used the topography analysis method.

In the Figure S4, we analyzed the twist angle and strain of the twisted hBN at several points. Figure S4b-S4e show the different twist angles from $\sim0.11^{\circ}$ to $\sim0.25^{\circ}$ because the moiré lengths are from ~ 58 nm to ~ 130 nm. So, even in the one twisted hBN sample, there is a local twist angle difference. Moreover, we assume that slight elongations from each moiré patterns formed due to local strains. If the twist and strain is on the moiré structure simultaneously, then the elongation of triangular shape moiré structures can be realized.

In addition, Figure S4f is a cropped KPFM image from Fig. 3f. to show the local strain difference. The moiré patterns can be classified as twist, isotropic strain, diagonal pure shear, and horizontal simple shear (HSS)^12^. Since the only case showing striped patterns is HSS, we apply this case to analyze this sample. For the HSS case, the relationship between moiré length and strain is $\lambda_{M}=a/\varepsilon$. So, we conclude the different strain from ~ 0.04 % to ~0.06% because its moiré lengths are from ~ 417 nm to ~ 626 nm.

**Supporting Note 6. Additional analysis for the potential depth in multiple moiré superlattice by the hBN thickness and moiré length.**

In Figure S5, there is a graph of the potential depth ($\Delta V$) by moiré length ($L_{M}$) with a same method of Fig. 1g in the main text. So, Fig. S5a. and S5b. are the graphs according to the equation (1) in the main text. Figure S5c. is the 3D schematic of the sample to visualize the multiple moiré interface structure. Figure S5d. is the same graph as Fig. 2h. showing the potential line profile from one interface and two interface systems.

According to Fig. S5c. and S5d., the three regions (A, B, C) have different polarization orientations. A and B have two moiré interfaces, and C has only one interface. When we set the tip-to-sample distance as $z= 20 nm$ for the C area, the distance in the A, B area become $z= 21.5 nm$ because of the additional top hBN’s thickness ($d_{T}=\sim1.5 nm$). So, we plot two graphs in Fig. S5b. for $z= 20 nm$ and $z= 21.5 nm$ to compare the two twist interfaces area (A, B) and one interface area (C).

**Table S2. Matching the KPFM data with fitting results**

|  | **A** | **B** | **C** |
| --- | --- | --- | --- |
| P | $\uparrow\uparrow,\uparrow\downarrow$ | $\downarrow\uparrow,\downarrow\downarrow$ | $\uparrow, \downarrow$ |
| $L_{M}$ | ~ 124 nm | ~ 101 nm | ~ 104 nm |
| $\Delta V$ | **~ 131.5 mV** | **~ 92.6 mV** | **~ 123 mV** |
| $\Delta V(20nm)$ | 152.1 mV | 116.5 mV | **121.3 mV** |
| $\Delta V(21.5nm)$ | **139.3 mV** | **104.6 mV** | 109.3 mV |

In Table S2, we put all the parameters like polarization (P), moiré length ($L_{M}$), and potential depth data ($\Delta V$) measured by KPFM in each area and compare it with calculation data ($\Delta V(20nm)$, $\Delta V(21.5nm)$). According to Table S2., potential depth in the A, B area (~ 135.5 mV, ~ 92.6 mV) is very similar with calculated data for $z= 21.5 nm$ (139.3 mV, 104.6 mV) and the potential depth from the C area (~ 123 mV) is also matched with $\Delta V\left( 20nm \right)$ (121.3mV).

The amplitude from A and C is not so different from each other. However, it is because the dominant parameter is moiré length not the thickness difference. In addition, the amount of step-like decrease in the B area is much larger than that of the A area. It is because the moiré size of A and B is different. As shown in Fig. 2g., the moiré pattern of B is much larger than A. So, we conclude the potential depth is much bigger in the B area because the moiré length is longer than the A area.

**Supporting Note 7. KPFM comparison of twisted hBN by exposing 532 nm laser.**

To confirm the effect of deep UV femtosecond laser on twisted hBN, we performed additional measurement with the continuous-wave green (532 nm) laser. In Figure S6, we obtained KPFM data before and after exposing the 532nm laser. According to the KPFM images in Fig. S6a and S6b, there is no significant change, and it is confirmed by line profiles in Fig. S6c and S6d. The Raman spectroscopy data in Fig. S6e. is obtained $\lambda=532nm$, P=20mW, 300g/mm, 1s at room temperature and ambient pressure. The result exhibits a clear hBN peak (~ 1369 ${cm}^{-1}$) in the sample. The additional measurement with 532nm laser strengthens our theory about the deep UV femtosecond laser’s effect on twisted hBN.

**Supporting Note 8. The method for fitting curve from surface potential difference by moiré length graph.**

According to the equation (1) in the main text, we can predict the electrostatic potential in twisted hBN. The vertical distance to the interface ($z=d_{T}$+ $z_{lift}$) can be calculated by the sum of top hBN thickness ($d_{T}$) and tip-to-surface distance ($z_{lift}$). So, the relation between potential depth and moiré length can be fitted by $\Delta V\sim exp(-\frac{4\pi z}{\sqrt{3}\lambda_{m}})$.

All the error bars in Fig. 1g. represent the root mean square error from the fitting of KPFM measurement. The detail calculation of the error bar is as follows

$$ERR\left( L_{M} \right)=\sqrt{MSE\left( L_{M} \right)}=\sqrt{\frac{1}{n}\sum_{i=1}^{n} {(V\left( L_{M} \right)-\hat{V}\left( L_{M} \right))}^{2}}$$

where the MSE is the mean squared error, $V$ is the observed potential depth values and $\hat{V}$ is the predicted values by equation (1).

When it comes to the lift height, we used a parameter of the lift height in the software as a reference (12.1 nm). The AFM probe oscillation amplitudes should be counted which are generally in the range of 1 nm or less in non-contact mode. So, we recalculated the fitting curve with the uncertainty of lift-height ($12.1 \pm1 nm$) and we draw the sky-blue lines around the blue line to see the range of uncertainty.

**Supporting Note 9. The time dependence results after exposing deep UV femtosecond laser.**

To see the time dependence of the deep UV femtosecond laser effect, we take KPFM data again after 81 days. In Fig. S7a. and S7b., the potential amplitude from line profile is decreased from $\pm50mV$ to $\pm30mV$ after exposing deep UV laser. After 81 days later, the potential is almost the same (Fig. S7c). Therefore, we confirm that the change of moiré potential by the irradiation from the femtosecond deep UV laser is not temporary.

**Supporting Note 10. The optimization of KPFM/PFM measurement.**

To obtain the state-of-art level KPFM and PFM results, we conducted all the measurements after accurate calibration to set proper amplitude and phase and frequency of AC voltage. In addition, we always optimize the drive amplitude and frequency of tip to select the exact cantilever’s resonant peak. Therefore, we could detect ± 20 ~ 400 mV potential modulation by moiré superlattice as the previous studies^13–15^. In our work, we use both frequency-modulation (FM-KPFM) and amplitude-modulation Kelvin probe force microscopy (AM-KPFM). According to the recent studies^13,16^, FM-KPFM has a higher spatial resolution than AM-KPFM. However, its signal-to-noise ratio is lower. According to the pros-and-cons, we used both AM-KPFM and FM-KPFM modes depending on the purpose of each measurement. So, all the KPFM data except Fig. 1 were obtained by AM-KPFM to enhance the signal-to-noise ratio and obtain clearer moiré pattern images.

**Figure captions**


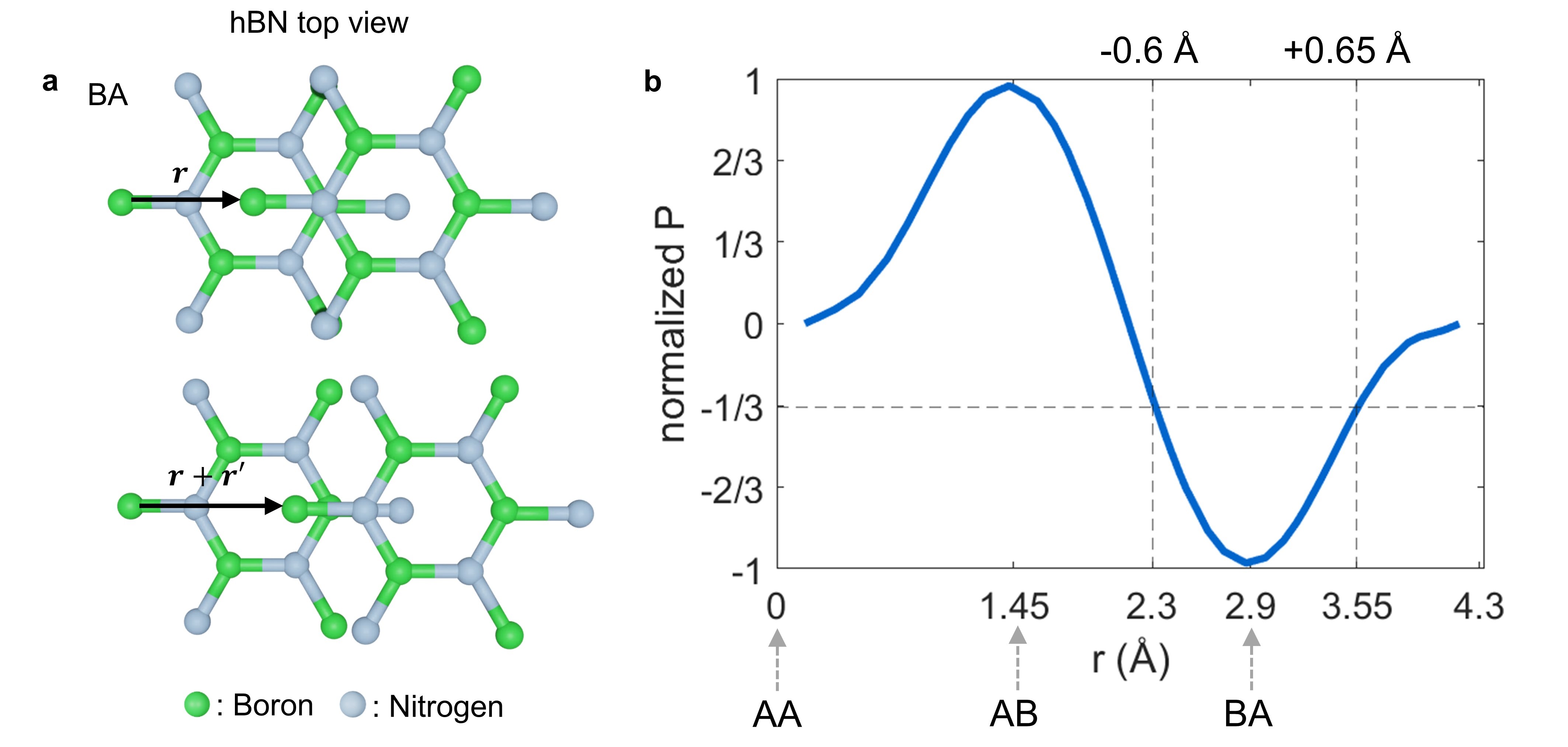


**Figure S1 | Electrical polarization as a function of in-plane displacement of twisted hBN. a,** hBN top view of BA staking and slightly misaligned configuration. In-plane displacement r means the displacement between two boron atoms in adjacent layers. So, AA staking has 𝑟 = 0. AB and BA staking has 𝑟 = 1.45 Å, 𝑟 = 2.9 Å respectively. **B,** Normalized polarization as a function of r from the equation (1). If there is in-plane displacement about $\pm0.6 \sim0.65 Å$ from BA stacking, the polarization is decreased ~ 1/3. So, the predicted displacement is about $\pm0.6 \sim0.65 Å$ for AB/BA stacking of twisted hBN after exposure to the femtosecond laser.


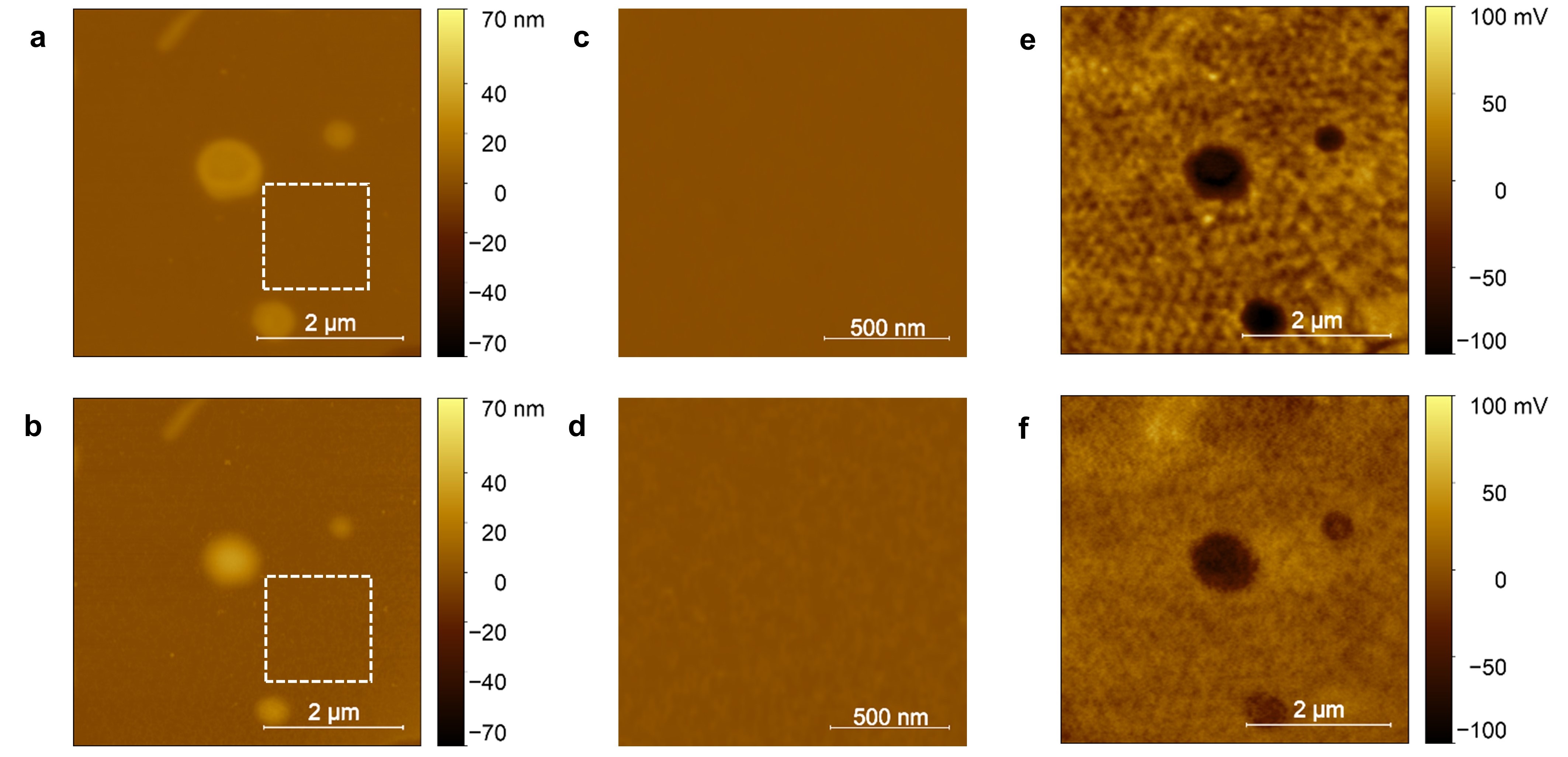


**Figure S2 | Topology and KPFM comparison to exclude any topological defect or doping effect by femtosecond laser.** **a,** AFM topography of twisted hBN sample in Fig. 4b of main text before deep UV femtosecond laser irradiation and **b,** after irradiation. **c-d**, cropped AFM topography from (**a)** and (**b)**. change After exposure to the laser, there are no holes or defects on the sample. **e,** KPFM image before and (**f)** after laser irradiation. The KPFM result shows no doping effect.

**
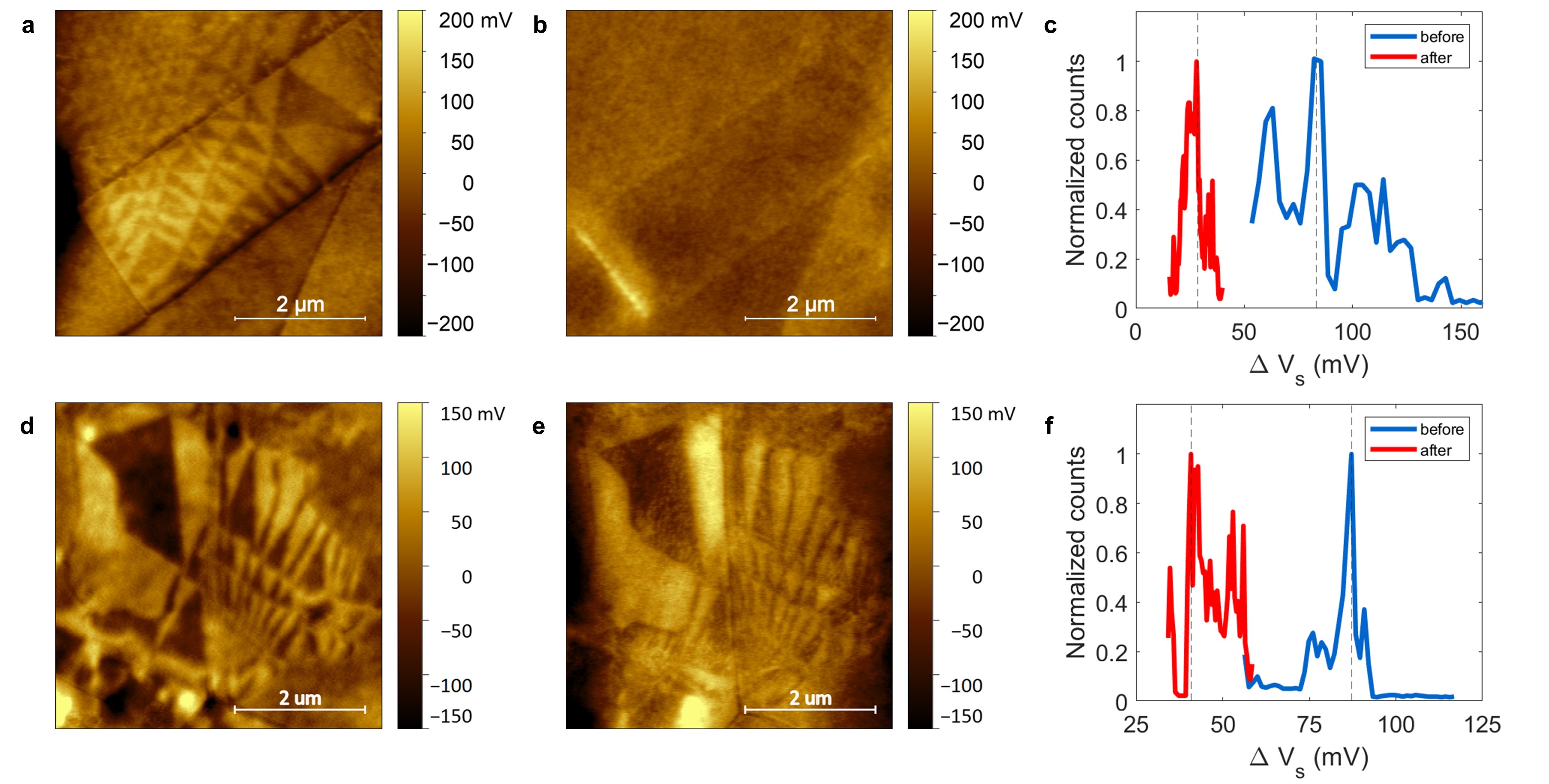
**

**Figure S3 | Additional statistical analysis of moiré depolarization by deep UV femtosecond laser.** **a,** AFM Topography image of twisted hBN sample in Fig. 4f and **(b)** its KPFM image. **c,** Statistical analysis of moiré potential before and after deep UV femtosecond laser irradiation. **D,** Topography and **(e)** KPFM image of another twisted hBN sample. **f,** Statistic result showing the significant depolarization after irradiation.


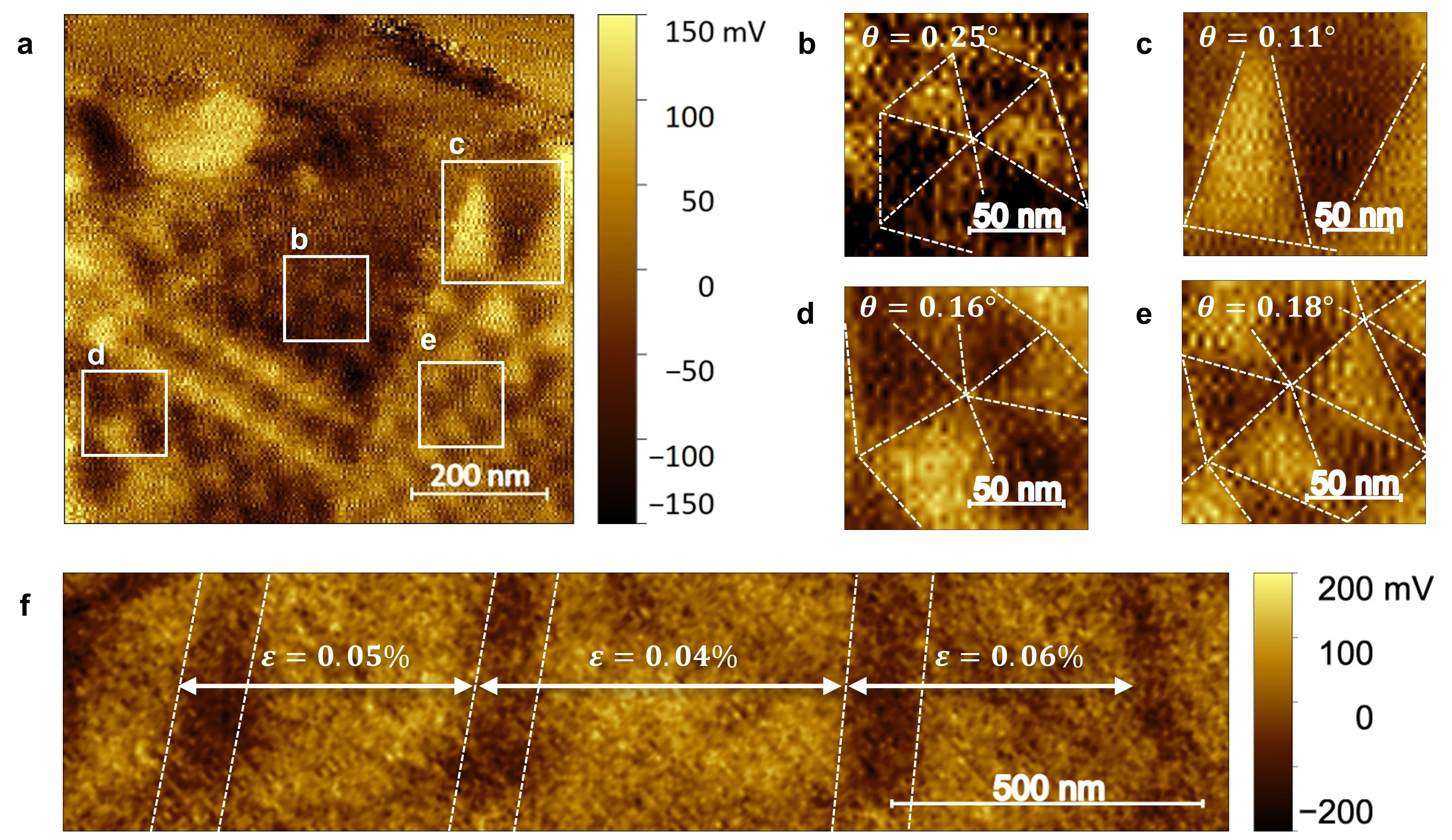


**Figure S4 | Twist angle and strain of twisted hBNs. a,** KPFM data of Fig. 2c. **b-e,** cropped images with different twist angles **(b)** $0.25^{\circ}$, **(c)** $0.11^{\circ}$, **(d)** $0.16^{\circ}$, **(e)** $0.18^{\circ}$. **f,** KPFM data from Fig. 3f showing the different shear strain 0.04 ~ 0.06 %.


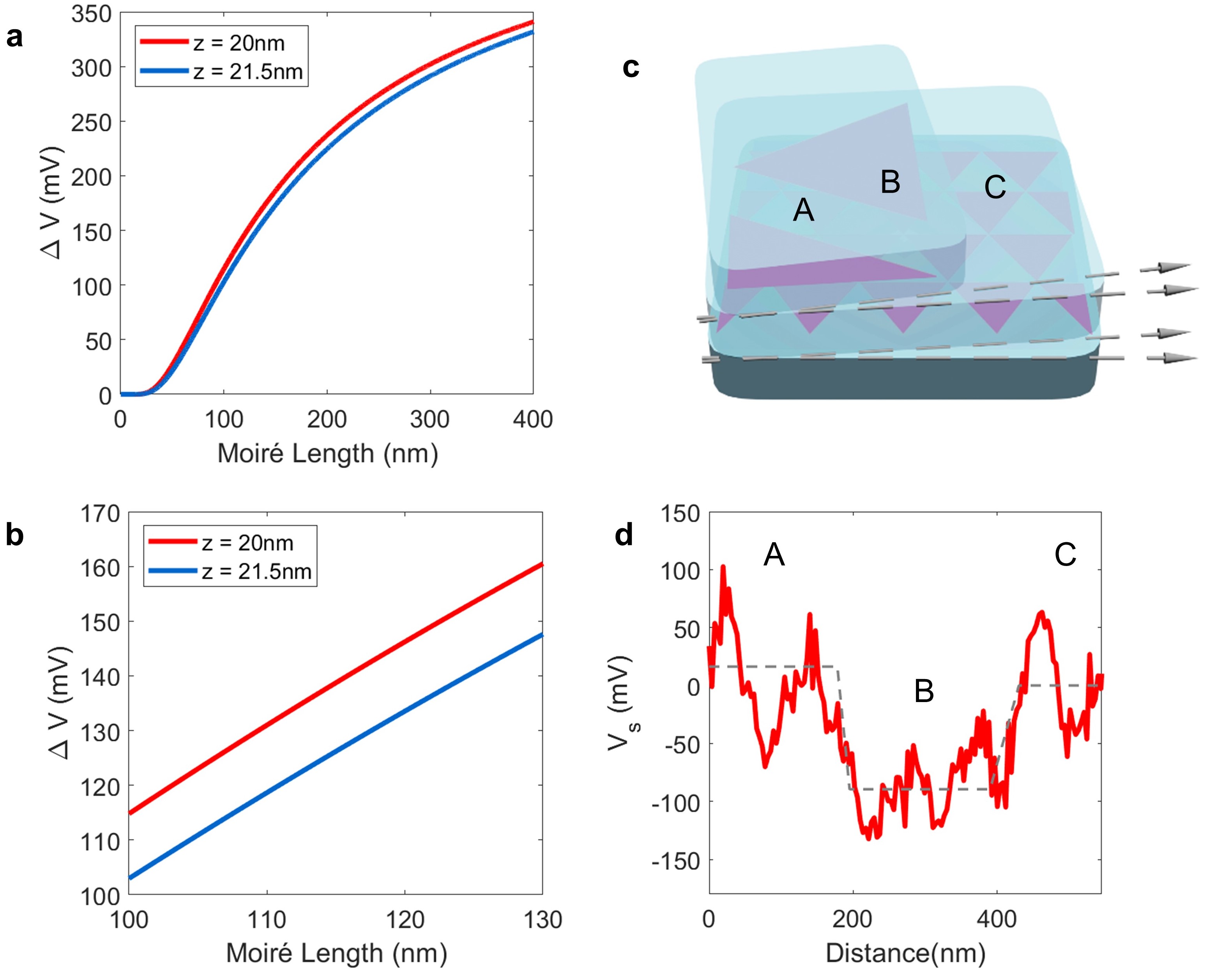


**Figure S5 | Potential depth by moiré length and tip-to-sample distance and the analysis of twisted hBN which has both single interface and two interfaces. a-b,** Potential depth fitting curve by different tip-to-sample distance. **c,** Schematic of the sample with single interface and two interfaces. **d,** KPFM line profile from the sample.


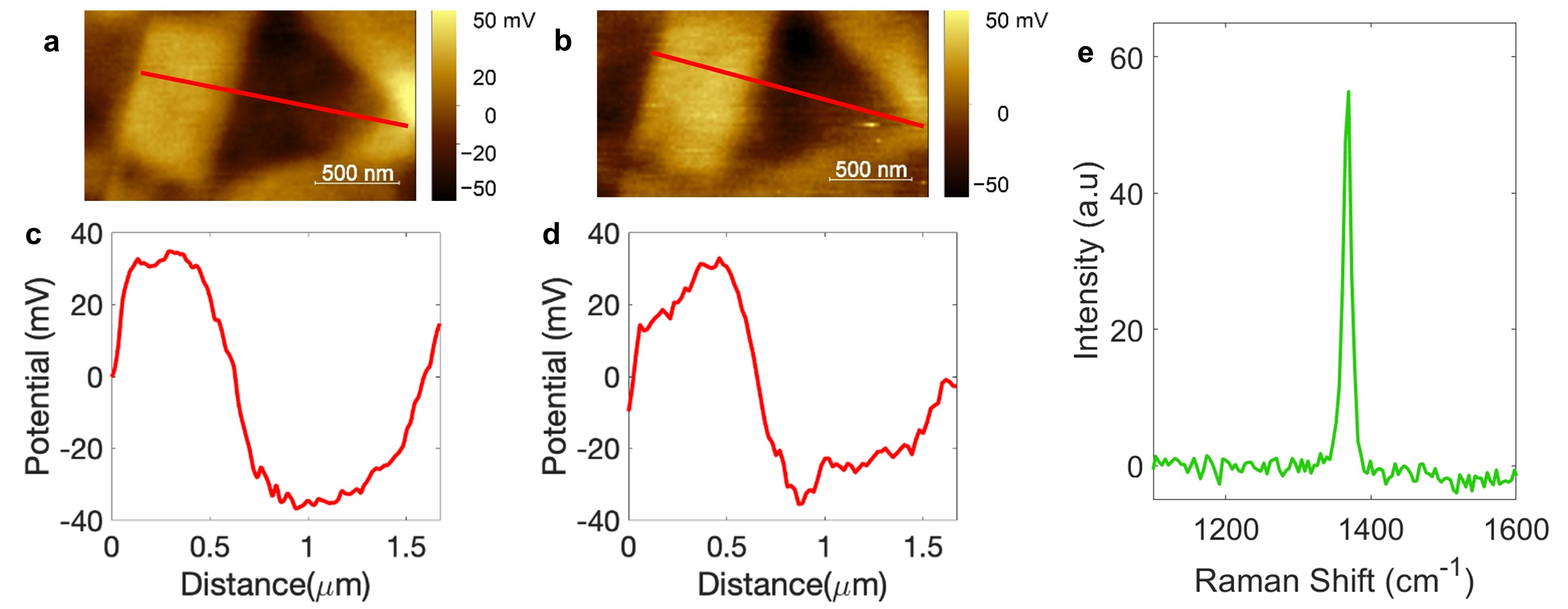


**Figure S6 | KPFM comparison of twisted hBN by exposing 532 nm laser. a,** KPFM image before and (**b)** after exposure to 532 nm laser. **c-d,** Potential line profiles from (**a)** and (**d)** showing no significant change. **e,** Raman spectroscopy data from the sample.

**
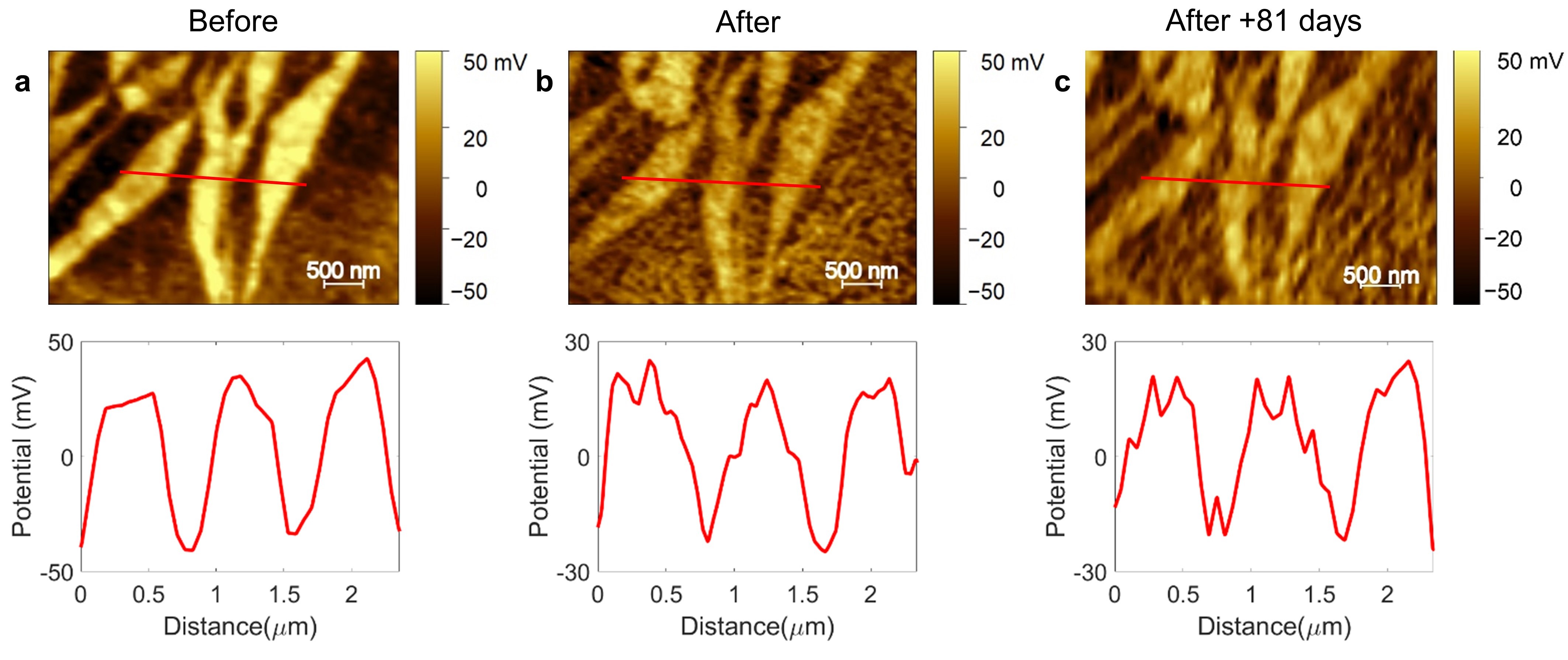
**

**Figure S7 | The time dependence results after exposing deep UV femtosecond laser. a,** KPFM image and its line profile before and (**b)** after exposure to deep UV femtosecond laser. **c,** the KPFM result after 81 days showing no significant change from **(b)**

**Supporting Information Reference**

1. Zhao, P., Xiao, C. & Yao, W. Universal superlattice potential for 2D materials from twisted interface inside h-BN substrate. *npj 2D Mater Appl* **5**, 1–7 (2021).

2. Li, L. & Wu, M. Binary Compound Bilayer and Multilayer with Vertical Polarizations: Two-Dimensional Ferroelectrics, Multiferroics, and Nanogenerators. *ACS Nano* **11**, 6382–6388 (2017).

3. Constantinescu, G., Kuc, A. & Heine, T. Stacking in Bulk and Bilayer Hexagonal Boron Nitride. *Phys. Rev. Lett.* **111**, 036104 (2013).

4. Ginsberg, J. S. *et al.* Phonon-enhanced nonlinearities in hexagonal boron nitride. *Nat Commun* **14**, 7685 (2023).

5. Tancogne-Dejean, N. & Rubio, A. Atomic-like high-harmonic generation from two-dimensional materials. *Science Advances* **4**, eaao5207 (2018).

6. Chen, C. Y. *et al.* Unzipping hBN with ultrashort mid-infrared pulses. *Science Advances* **10**, eadi3653 (2024).

7. *Kelvin Probe Force Microscopy: Measuring and Compensating Electrostatic Forces*. vol. 48 (Springer, Berlin, Heidelberg, 2012).

8. *Scanning Probe Microscopy: Electrical and Electromechanical Phenomena at the Nanoscale*. (Springer, New York, NY, 2007). doi:10.1007/978-0-387-28668-6.

9. Xiong, X. *et al.* Oxygen Incorporated MoS2 for Rectification-Mediated Resistive Switching and Artificial Neural Network. *Advanced Functional Materials* **34**, 2213348 (2024).

10. Choi, M. S., Lee, M., Ngo, T. D., Hone, J. & Yoo, W. J. Chemical Dopant-Free Doping by Annealing and Electron Beam Irradiation on 2D Materials. *Advanced Electronic Materials* **7**, 2100449 (2021).

11. Atri, S. S. *et al.* Spontaneous Electric Polarization in Graphene Polytypes. *Advanced Physics Research* **3**, 2300095 (2024).

12. Cazeaux, P., Clark, D., Engelke, R., Kim, P. & Luskin, M. Relaxation and Domain Wall Structure of Bilayer Moiré Systems. *J Elast* **154**, 443–466 (2023).

13. Kim, D. S. *et al.* Electrostatic moiré potential from twisted hexagonal boron nitride layers. *Nat. Mater.* **23**, 65–70 (2024).

14. Woods, C. R. *et al.* Charge-polarized interfacial superlattices in marginally twisted hexagonal boron nitride. *Nat Commun* **12**, 347 (2021).

15. Vizner Stern, M. *et al.* Interfacial ferroelectricity by van der Waals sliding. *Science* **372**, 1462–1466 (2021).

16. Zerweck, U., Loppacher, C., Otto, T., Grafström, S. & Eng, L. M. Accuracy and resolution limits of Kelvin probe force microscopy. *Phys. Rev. B* **71**, 125424 (2005).
